# Case Report: Infantile-Onset Fulminant Type 1 Diabetes Mellitus Caused by Novel Compound Heterozygous *LRBA* Variants

**DOI:** 10.3389/fimmu.2021.677572

**Published:** 2021-04-12

**Authors:** Eriko Totsune, Tomohiro Nakano, Kunihiko Moriya, Daichi Sato, Dai Suzuki, Akinobu Miura, Saori Katayama, Hidetaka Niizuma, Junko Kanno, Menno C. van Zelm, Kohsuke Imai, Hirokazu Kanegane, Yoji Sasahara, Shigeo Kure

**Affiliations:** ^1^ Department of Pediatrics, Tohoku University Graduate School of Medicine, Sendai, Japan; ^2^ Department of Immunology and Pathology, Monash University and Alfred Hospital, Melbourne, VIC, Australia; ^3^ The Jeffrey Modell Diagnostic and Research Centre for Primary Immunodeficiencies, Faculty of Medicine, Nursing & Health Sciences, Monash University, Melbourne, VIC, Australia; ^4^ Department of Community Pediatrics, Perinatal and Maternal Medicine, Tokyo Medical and Dental University, Tokyo, Japan; ^5^ Department of Child Health and Development, Graduate School of Medical and Dental Sciences, Tokyo Medical and Dental University (TMDU), Tokyo, Japan

**Keywords:** infantile-onset fulminant type 1 diabetes mellitus, LRBA deficiency, refractory autoimmune cytopenia, CTLA-4 deficiency, transposable elements (TE)

## Abstract

Lipopolysaccharide-responsive beige-like anchor (LRBA) deficiency is a subtype of common variable immune deficiency (CVID). Numerous case reports and cohort studies have described a broad spectrum of clinical manifestations and variable disease phenotypes, including immune dysregulation, enteropathy, and recurrent infections. Although LRBA deficiency is an autosomal recessive primary immunodeficiency resulting in a phenotype similar to CVID, it is a monogenic disease and separate from CVID. Recently, in a report of monogenic primary immunodeficiency disorder associated with CVID and autoimmunity, the most common mutated gene was *LRBA*. We report the case of a girl who presented with fulminant type 1 diabetes at age 7 months. She later experienced recurrent bacterial infections with neutropenia and idiopathic thrombocytopenic purpura. Clinical genome sequencing revealed compound heterozygosity of the *LRBA* gene, which bore two novel mutations. A genetic basis should be considered in the differential diagnosis for very young patients with fulminant autoimmunity, and the diagnostic work-up should include evaluation of markers of immunodeficiency.

## Introduction

Lipopolysaccharide-responsive beige-like anchor (LRBA) deficiency was first described in 2012 as an autosomal-recessive disorder caused by biallelic mutations in the *LRBA* gene (OMIM #614700). It was initially characterized as the cause of early-onset hypogammaglobulinemia and autoimmune manifestations and as imparting susceptibility to inflammatory bowel disease and recurrent infection (1). The major autoimmune components involved includes enteropathy and cytopenia. Fulminant type 1 diabetes (FT1DM) is a less frequently reported autoimmune manifestation of LRBA with minimal details regarding the clinical features and characteristics (1). LRBA deficiency seriously interferes with the intracellular trafficking of cytotoxic T-lymphocyte protein-4 (CTLA-4), rerouting the protein away from lysosomal degradation and back to the cell surface (2). CTLA-4 is a key immune checkpoint protein that is constitutively expressed on fork-head box P3^+^ (FoxP3^+^) regulatory T-cells and is also induced following activation of conventional T-cells (3). LRBA deficiency results in low CTLA-4 expression, which explains the phenotypic overlap between LRBA- and CTLA-4-deficient patients (4, 5).

To date, various agents have been used to treat LRBA deficiency, including corticosteroids, intravenous immunoglobulins, sirolimus, infliximab, rituximab, and azathioprine (6–9). Some patients also benefit from hematopoietic stem cell transplantation (HSCT), which can be curative (6, 9). More recently, abatacept, a CTLA-4–immunoglobulin fusion protein, has been reported to control disease-related immune dysregulation phenotypes (10). Here, we describe a patient presenting with a combination of FT1DM and refractory autoimmune cytopenia and harboring a novel compound heterozygous mutation in *LRBA*.

## Materials and Methods

### Study Approval

This study was conducted in accordance with the Helsinki Declaration and approved by the Ethics Committee of the Tohoku University (2019-1-561).

### Genetic Analysis

Genomic DNA was extracted from a blood sample by using PAXgene Blood DNA system (Becton, Dickinson and Company) according to manufacturer’s instructions. The PCR primers for *LRBA* exon 12 were 5′- TGTTGGAAGCAGTTTTAGTGGA -3′ and 5′- GAGGAATGGAGGCAAGGTAA -3′. The PCR primers for defining the exon 35-41 deletion were 5′- AGTTGGTTACTTGATAGGGCTG -3′ and 5′- CCAGAGCCATGGGTACATTTAG -3′. Total RNA was extracted from a blood by using QIAamp RNA blood mini kit (QIAGEN) and cDNA was generated by PrimeScript™ RT reagent Kit with gDNA Eraser (TaKaRa). The RT-PCR primers designed on exon-exon junctions were 5′- GAAGGAACAAGTCTGGTTTGC -3′ and 5′- TGAACATCACAGCAACTCTG -3′. DNA sequencing was performed with by standard capillary methodology using the same primers as for PCR with the addition of the primer to identify the genomic breakpoint of *LRBA*: 5′- CTCAATCCCACTTTGCTGA -3′.

The genomic sequence of *LRBA* including the 10 kb upstream and downstream sequences (771,293 bp) was extracted from Ensembl v103 – Feb 2021. The complete *LRBA* gene sequence and the 2000bp flanking both 5’ and 3’ breakpoints were annotated with TE-derived interspersed repeats by the CENSOR software tool of the Repbase database as described before (11).

### Cell Isolation, Staining and Flow Cytometric Analysis

Whole blood samples were collected from the patient and controls. Peripheral blood mononuclear cells (PBMCs) were isolated from whole blood by using Lymphoprep (Axis Shield Diagnostics Ltd., Dundee, Scotland) gradient centrifugation according to the manufacturer’s instructions. The cells were washed twice and resuspended in phosphate-buffered saline (PBS). 1,000,000 PBMCs were stained with each cocktail containing the monoclonal antibodies previously described (12).

For evaluation of CTLA-4 expression, PBMCs were stimulated at 37°C for 16 hours with anti-CD3/CD28 activating Dynabeads (Life Technologies, Oslo, Norway). After the beads were removed by magnetic separation, cells were stained with PC7-conjugated anti-CD4 (clone, SFCI12T4D11 (T4), Beckman Coulter) at 4°C for 15 min. Cells were fixed and permeabilized with Fixation/Permeabilization kit (eBioscience) at 4°C for 30 min followed by 2 wash steps with permeabilization buffer (eBioscience). Cells were stained with Alexa Fluor 647-conjugated anti-FOXP3 (clone 236A/E7, eBioscience) and PE-Cy5-conjugated CTLA4 (clone BNI3, BD Biosciences) mAbs at 4°C for 20 min as previously described (13). After washing with permeabilization buffer (eBioscience), stained cells were analyzed by flow cytometer. All flowcytometry was performed using a BD LSR Fortessa flow cytometer (Becton Dickinson, Franklin Lakes, NJ) and data were analyzed with FlowJo 10.1r5 analysis software (FlowJo, LLC, Ashland, OR).

### Evaluation of LRBA Protein Expression by Western Blotting

PBMCs were lysed in buffer (CellLytic M, Sigma-Aldrich) containing protease inhibitor cocktail (Sigma-Aldrich). Equal amounts of protein, according to Pierce™ BCA Protein Assay Kit (Thermo Fisher Scientific), were resolved by sodium dodecyl sulfate-polyacrylamide gel electrophoresis in a Mini-PROTEAN^®^ TGX™ 7.5% Precast Gel (Bio-Rad) and transferred to a polyvinylidene difluoride membrane. The membrane was probed using an antibody to LRBA (HPA023597, Sigma-Aldrich) or β-actin (017-24551, Wako). The appropriate horseradish peroxidase-conjugated secondary antibody was incubated with the membrane, and antibody binding was detected using the Amersham ECL Prime Western Blotting Detection Reagents (GE Healthcare) and ChemiDoc MP system (Bio-Rad).

## Results

### Case Presentation

**Figure 1:**
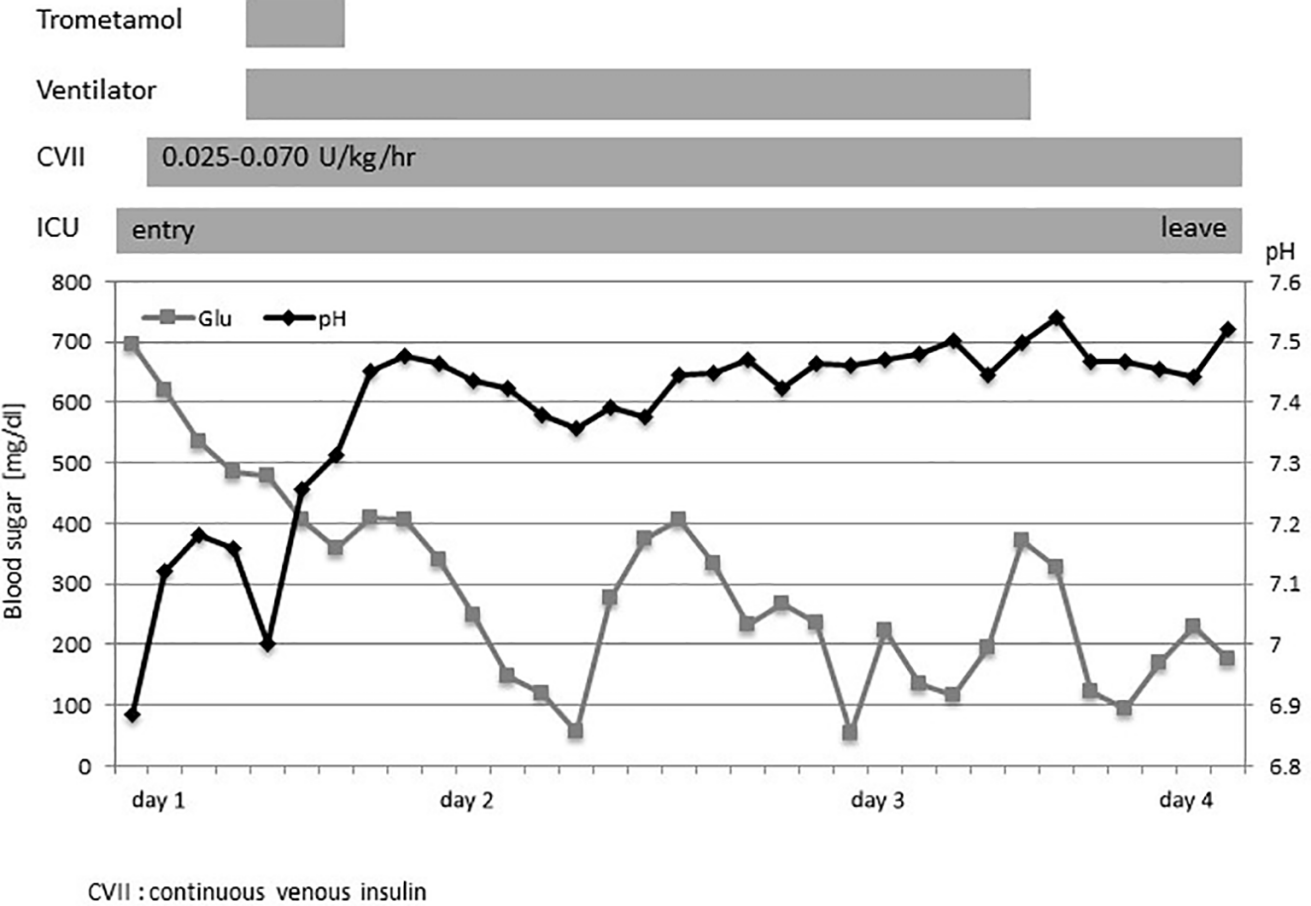
The clinical course at admission. The clinical course and changes in the blood sugar level (◼) and pH (◆). CVII, continuous venous insulin; ICU, intensive care unit.

The patient was born at term as the second child of nonconsanguineous Japanese parents. She developed FT1DM at the age of 7 months; the initial symptoms included fever and vomiting. The laboratory findings indicated pronounced ketoacidosis (pH of 6.884, pCO_2_ level of 39.6 mmHg, base excess of −25.1 mmol/L, and ketone level of 12,315 mmol/L), a blood glucose level of 695 mg/dL, and an HbA1c level of 6.4% (**Supplementary Table 1**). The patient was positive for insulin autoantibodies (> 5,000 nU/mL) but negative for glutamate decarboxylase autoantibodies (< 5.0 U/mL). As she had difficulty breathing, she was admitted to the intensive care unit. Initial fluid replacement with saline, followed by continuous intravenous insulin infusion (0.025–0.070 U/kg/h) did not improve the metabolic acidosis, prompting trometamol administration and mechanical ventilation support. The metabolic acidosis, hyperglycemia, and ketosis then improved (**Figure 1**). In a glucagon test, the connecting peptide immunoreactivity was <0.03 ng/mL both before and after glucagon loading, suggesting that insulin secretion was depleted. At initial discharge, she was provided with a sensor-assisted pump (insulin 0.15–0.10 U/h), and the blood glucose level was well-controlled. Two months later, she developed mandibular cellulitis, and her neutrophil count was zero. Bone marrow examination revealed hyperplasia without impaired differentiation of myeloid cells. High serum levels of autoantibodies against human neutrophil antigen-1a and -1b were detected, leading to a diagnosis of infantile immune neutropenia. Antibiotic prophylaxis was commenced.

### Development of Autoimmune Disease Presentation

When she was 13 months of age, she developed immune thrombocytopenia. She was repeatedly given intravenous immunoglobulin to prevent severe thrombocytopenia. Additionally, serum amylase and lipase levels were elevated, but abdominal ultrasonography revealed no obvious pancreatitis. Contrast-enhanced computed tomography revealed inflammation in the lower lobes of lung, splenomegaly, and lymphadenopathy in the right deep neck, lower left jaw, bilateral axillae, and para-aortic lymph nodes, but no evidence of hepatitis or pancreatitis. Laboratory tests revealed the absence of antinuclear and anti-double-stranded DNA autoantibodies. Of note, the soluble interleukin-2 receptor level was markedly elevated (4,677 U/mL). Immunological studies performed at that time revealed essentially normal lymphocyte counts, although activated T-cells were increased and memory B-cells decreased in number (**Supplementary Table 2**). Subsequent magnetic resonance imaging revealed neither intracranial nor eye lesions. The patient was commenced on 5 mg/kg/day oral prednisone at the age of 16 months, and her platelet count has been maintained at ~80,000/μL.

### Compound Heterozygous Mutations in LRBA Detected by Clinical Sequencing Panel

**Figure 2:**
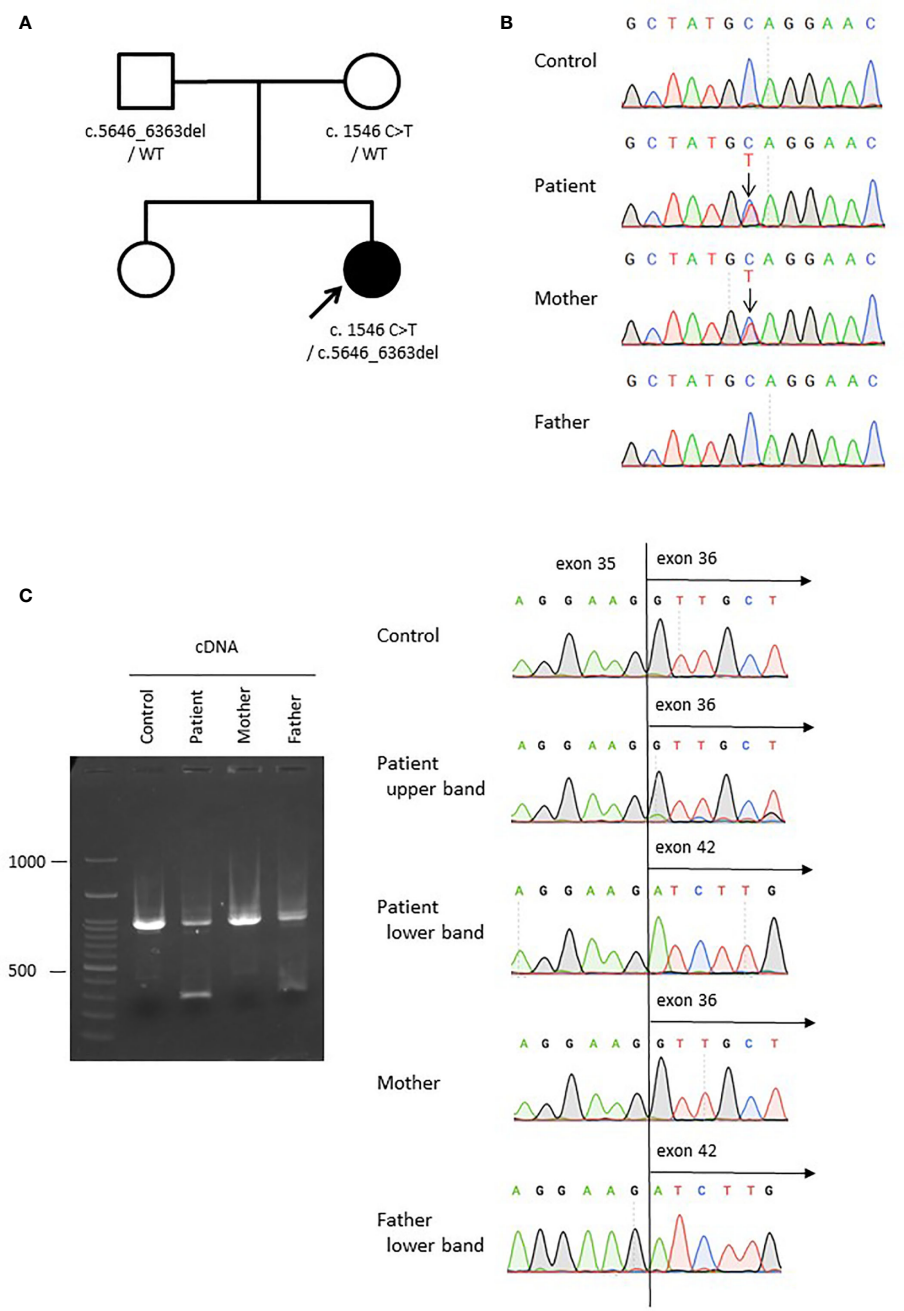
Compound heterozygous germline *LRBA* mutations. **(A)** The pedigree of the family. **(B)** Sanger sequencing of genomic DNA from the patient, mother, and father. A change from C to T was detected (c.1546C>T, p.Gln516) in the patient and her mother. **(C)** Sanger sequencing of *LRBA* cDNA from the patient, mother, and father, showing a large deletion from exon 36 to 41 (c.5646_6363del) in the patient and her father. The *LRBA* cDNA sizes are 988 bp for the wild-type allele but 270 bp for the mutant allele lacking exons 36–41.

**Figure 3:**
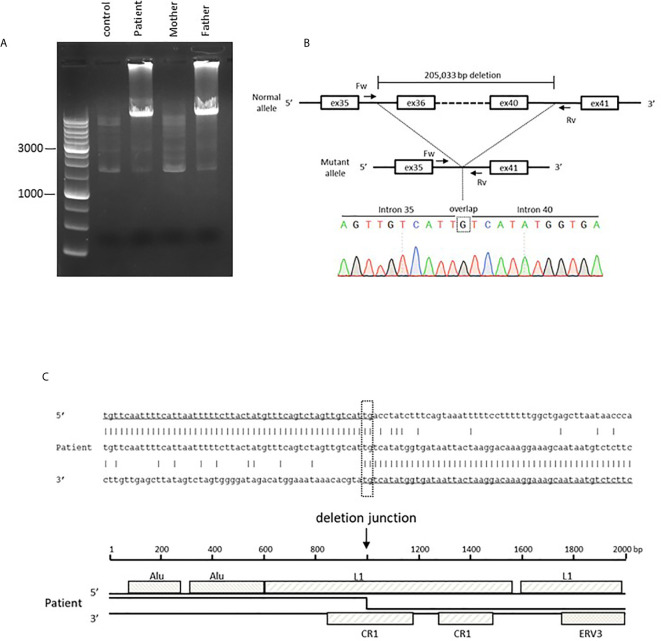
Genetic analysis of a large heterozygous deletion in the *LRBA* gene. **(A)** Electrophoresis of the PCR product harboring the large deletion. The wild-type allele is too large for amplification. **(B)** Sanger sequencing of the breakpoint region. The 5′ breakpoint is located at chr4: 150,736,531 and the 3′ breakpoint at chr4:150,531,499 (GRCh38/hg38). This includes a one-nucleotide (G) overlap. **(C)** The sequence of the mutant allele and the reference sequences of the 5′ and 3′ breakpoint regions. The breakpoint region includes 2 bp (TG) of microhomology. Transposable element analysis showed that both breakpoints were located within LINEs. The 5′ breakpoint was within LINE1 (L1) and the 3′ breakpoint within Chicken repeat 1 (CR1).

Given the multiple autoimmune manifestations, autoimmune lymphoproliferative syndrome was suspected. Targeted clinical sequencing of 18 genes known to cause autoimmune lymphoproliferative syndrome (The Twist BioScience custom targeted panel, Illumina NextSeq) was performed in blood samples and revealed two heterozygous mutations in *LRBA*. The first was a heterozygous nonsense mutation in *LRBA* (c.1546C>T, p.Gln516*, **Figures 2A, B**) inherited from the mother. The second was a large deletion involving exons 36–42. To confirm this deletion and determine the inheritance, we performed RT-PCR using primers targeting *LRBA* exons 35 and 42 and, as the template, total RNA prepared from the blood of the patient, mother, and father; the products were evaluated by electrophoresis and Sanger sequencing (**Figure 2C**). In all three individuals, the result was amplification of the expected 988-bp fragment, which included the wild-type sequence spanning exons 35–42. In addition, a 270-bp fragment was observed in the patient and her father. Sequencing revealed direct splicing of exon 35 to exon 42 and the absence of exons 36–41 (**Figure 2C**). The large deletion resulted in a frameshift and a stop at codon 42 (c. 5646_6363del, p.Leu1883Serfs*42). To identify the genomic breakpoints of the large deletion, PCR using a forward primer targeting intron 35 and a reverse primer targeting intron 41 successfully amplified the breakpoint region, and Sanger sequencing revealed a 205,033 bp deletion (**Figures 3A, B** **)**. Breakpoint sequence analysis revealed 2 bp of microhomology at the fusion site. Further analysis of transposable elements (TEs) demonstrated that both the 5′ and 3′ breakpoints were located within long interspersed elements (LINEs) (**Figure 3C**).

### Functional Deficiency of LRBA

**Figure 4:**
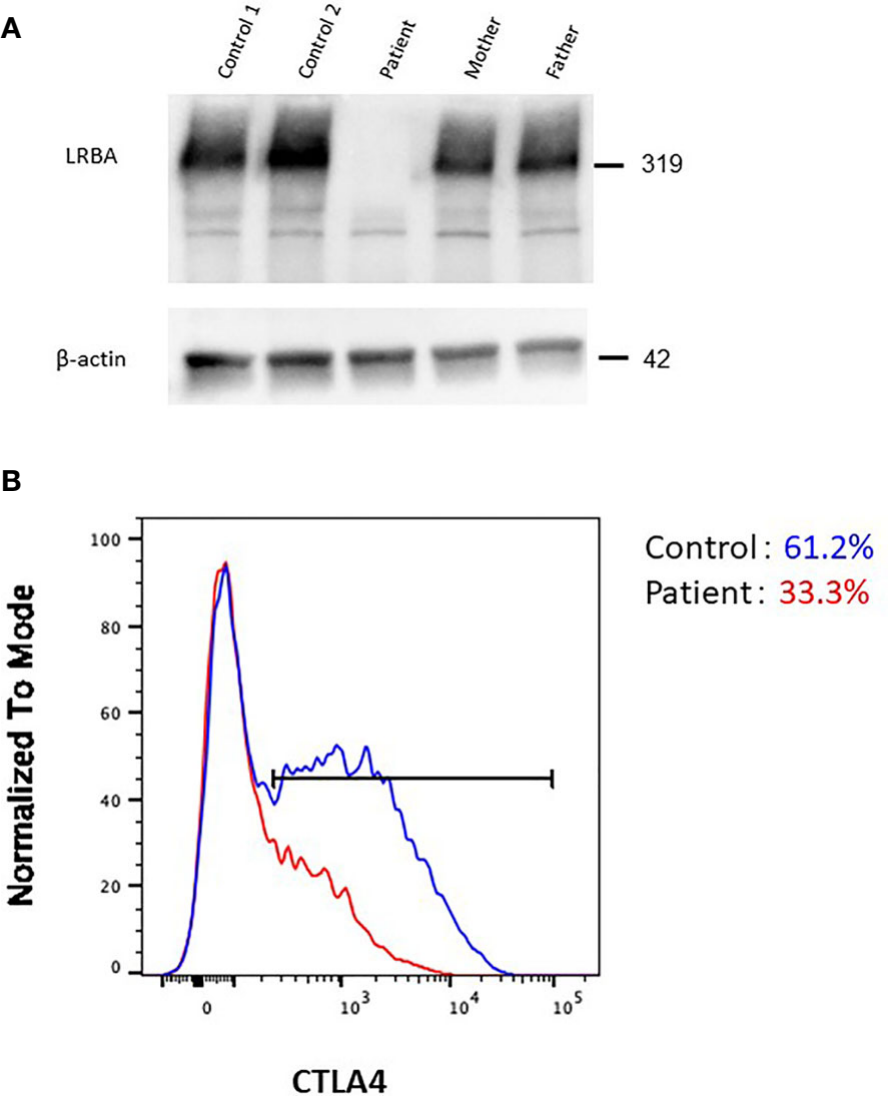
Effect of the pathogenic *LRBA* mutations on the protein level. **(A)** Immunoblotting of LRBA in T-cells from the patient (Pt), her heterozygous mother, her heterozygous father, and two independent healthy controls. The results are representative of those of two independent experiments. **(B)** Flow cytometric analysis of CTLA4 expression in CD4^+^FOXP3^+^ T-cells from a healthy control (blue line) and the patient (red line).

To determine whether the genetic lesions were disease-causing, the LRBA protein level was determined by immunoblotting. No LRBA protein was detected in T-cells from the patient (**Figure 4A**). Next, we analyzed the cellular CTLA4 protein level by flow cytometry. As reported previously, LRBA deficiency decreased CTLA4 expression compared with controls (**Figure 4B**), explaining the phenotypic overlap between LRBA- and CTLA4-deficient patients.

## Discussion

The LRBA protein regulates the expression of CTLA-4, which is a potent immune checkpoint receptor expressed by activated and regulatory T-cells. CTLA-4 blocks the stimulation/proliferation of T-cells and modulates immune responses (4, 14). Low expression of CTLA-4 in patients with LRBA deficiency results in partial loss of the regulatory effects on T-cell activation, leading to increased but inappropriate activation of T- and B-cells with impaired immune surveillance. This increases the risks of cancer and autoimmunity (1, 15). As the number of cases and cohorts grows, LRBA deficiency has been shown to exhibit a highly variable phenotypic presentation (9), including immune dysregulation, polyautoimmunity, organomegaly, and recurrent infections. A systematic review of 109 LRBA deficient patients found that 24% of them had insulin-dependent diabetes mellitus (IDDM). They report that the most frequent autoimmune complications in patients with LRBA deficiency were autoimmune hemolytic anemia (AIHA), idiopathic thrombocytopenic purpura (ITP), IDDM, and inflammatory bowel disease (IBD) (16). FT1DM is a less frequently reported autoimmune manifestation of LRBA deficiency, and few details on the clinical features and characteristics are available (6, 9). Recent studies suggest that FT1DM is common in east Asian countries including Japan, Korea, and China. In these countries, most FT1DM patients are adults; there are only a few pediatric reports (17–19). Early studies indicated no evidence of islet autoimmunity, but recent findings have increasingly suggested that islet autoimmunity is in fact involved in disease development (20). Gu et al. found β-cell autoantibodies (including anti-glutamate decarboxylase autoantibodies) in 2 patients, insulin cell autoantibodies in 1 patient, and islet cell autoantibodies in 1 patient among 23 FT1DM patients (17). T-cell mediated autoimmunity plays a role in the destruction of pancreatic β-cells (18). It is possible that β-cell-specific Th1 immunity, together with low-grade humoral immune responses, predisposes patients to FT1DM development. As in our patient, CTLA-4 expression was significantly reduced in the CD4^+^ helper T-cells of patients with FT1DM (21). In addition, genetic predispositions, especially involving the HLA-DQ and HLA-DR genes, have been associated with an increased susceptibility to FT1DM (11). Although the clinical course of pediatric FT1DM is similar to that of adults, the genetic background and mechanisms underlying the susceptibility to FT1DM may differ in pediatric patients, especially infants, as in our patient.

Our patient exhibited a large heterozygous deletion in *LRBA*; such deletions are a relatively frequent cause of LRBA deficiency (9). However, in previously described LRBA-deficient patients, the breakpoint regions were not determined. Large deletions in *IGHM*, *BTK*, and *DCLRE1C* (encoding Artemis) were shown to be associated with TEs (22), and both breakpoints in our patient were located within LINEs. *IGHM* and *DCLRE1C* are characterized by a high proportion of TEs (above the average in the human genome) associated with a high frequency of large deletions (22). The TE rate in *LRBA* is 43.0% (https://www.girinst.org/censor/), thus higher than the average (37%), perhaps increasing the risk of gross deletions. Also, *LRBA* is located on chromosome 4q31.3 within a recombination hotspot characterized by subtelomeric repetitive sequences. Such locations may exhibit large, intragenic germline deletions (22).

All patients with LRBA deficiency should be given immunoglobulin replacement therapy and prophylactic antibacterial therapy after molecular diagnosis. In fact, our patient has remained in good condition (without infection) on prophylactic antibacterial therapy. The currently available immunomodulatory therapies include corticosteroids, cyclosporine, mycophenolate mofetil, azathioprine, and rituximab. However, remission is achieved in only a subset of patients; large doses of steroids are often required to control active autoimmunity (6–9). Therapeutic approach by sirolimus for treatment of inflammatory and autoimmune disorder, and enteropathy in patients with LRBA deficiency was reported (7). Recently, abatacept, a T-cell modulator, has been proposed as a targeted precision therapy for LRBA-deficient patients (10). In addition, biomarkers including soluble CD25 and those of circulating follicular T-helper cells are useful for monitoring disease activity (10). It is unclear whether to proceed to HSCT, as the disorder is not always life-threatening. Recent findings suggest that the highest-risk patients are completely deficient in LRBA and eventually develop uncontrolled pulmonary disease, experiencing the worst disease outcomes (9). These findings call into question the prior recommendation to consider HSCT only in cases with severe phenotypes of LRBA deficiency, indicating that transplantation should be considered before the disease progresses. Although our patient remains well at 6 months after starting low-dose corticosteroids, further studies in a larger number of patients are required.

## Data Availability Statement 

The datasets presented in this study can be found in online repositories. The names of the repository/repositories and accession numbers can be found in the article/**Supplementary Material**.

## Ethics Statement

The studies involving human participants were reviewed and approved by the Ethics Committee of the Tohoku University. Written informed consent to participate in this study was provided by the participants’ legal guardian/next of kin.

## Author Contributions

ET, KM, DSa, DSu, AM, SKa and HN provided clinical information. ET, TN and KM wrote the manuscript. ET and TN performed the genetic and immunoblot analysis. KI and HK performed the flowcytometric analysis. HN, JK, HK, YS and SKu provided critical discussion. MZ performed bioinformatics analysis and edited the manuscript. KM supervised the study and edited the manuscript. All authors contributed to the article and approved the submitted version.

## Funding

KM was supported by grant from the Japanese Ministry of Health, Labor and Welfare of Japan (Grant Number 19K23819). MZ was supported by the Australian National Health and Medical Research Council (NHMRC; Senior Research Fellowship 1117687) and the Jeffrey Modell Foundation.

## Conflict of Interest

The authors declare that the research was conducted in the absence of any commercial or financial relationships that could be construed as a potential conflict of interest.
