# Supplementary material for: Case Report: Infantile-Onset Fulminant Type 1 Diabetes Mellitus Caused by Novel Compound Heterozygous LRBA Variants
Source: Front Immunol. 2021 Apr 12;12:677572. doi: 10.3389/fimmu.2021.677572 (PMC8072023; doi:10.3389/fimmu.2021.677572)
Supplement: Supplementary file 1 [file DataSheet_1.docx]

Supplementary material for " **Infantile-onset fulminant type 1 diabetes mellitus caused by novel compound heterozygous *LRBA* variants**" in the Frontier in Immunology

Eriko Totsune, Tomohiro Nakano, Kunihiko Moriya, Daichi Sato, Dai Suzuki,

Akinobu Miura, Saori Katayama, Hidetaka Niizuma, Junko Kanno, Menno C. van Zelm, Kohsuke Imai, Hirokazu Kanegane, Yoji Sasahara, Shigeo Kure

Corresponding author:

Kunihiko Moriya MD, PhD.

Department of Pediatrics, Tohoku University Graduate School of Medicine, 1-1 Seiryo-machi, Aoba-ku, Sendai 980-8574, Japan

E-mail: [kunihiko.moriya.a3@tohoku.ac.jp](mailto:kunihiko.moriya.a3@tohoku.ac.jp)

**Supplementary Table 1.** **Clinical data on admission**

| WBC | 42900 | /μl | T-Bil | 0.2 | mg/dl | IP | 6.5 | mg/dl | pH | 6.884 |  |
| --- | --- | --- | --- | --- | --- | --- | --- | --- | --- | --- | --- |
| Neut | 75.5 | % | γ-GTP | 17 | mg/dl | T-Cho | 156 | mg/dl | pCO2 | 39.6 | mmHg |
| Lymp | 19.5 | % | AST | 32 | U/l | TG | 497 | mg/dl | HCO3- | 7.1 | mmol/l |
| Hb | 10.6 | g/dl | ALT | 28 | U/l | CRP | 0.26 | mg/dl | BE | -25.1 | mmol/l |
| Plt | 57.7*10^4^ | /μl | LDH | 713 | U/l | Glu | 493 | mg/dl | Glu | 695 | mg/dl |
|  |  |  | AMY | 14 | U/l | HbA1c | 6.4 | % | Lac | 5.2 | mmol/l |
| PT-INR | 1.15 |  | BUN | 33 | mg/dl | Acetoacetic acid | 3766 | μmol/l |  |  |  |
| APT | 28.5 | sec | Cr | 0.34 | mg/dl | 3 hydroxybutyric acid | 8549 | μmol/l |  |  |  |
| FBG | 96 | mg/dl | UA | 18.7 | mg/dl | ketone bodies | 12315 | μmol/l |  |  |  |
| FDP | <2.5 | μg/ml | ALB | 4.0 | g/dl | Glucose albumin | 22.0 | % |  |  |  |
| D-dimer | 0.6 | μg/ml | Na | 141 | mEq/l | Insulin antibody binding rate | 70.8 | % |  |  |  |
|  |  |  | K | 5.3 | mEq/l | Insulin-autoantibody | >5000 | nU/ml |  |  |  |
|  |  |  | Cl | 109 | mEq/l | Anti-GAD antibody | <5.0 | U/ml |  |  |  |
|  |  |  | Ca | 9.5 | mg/dl |  |  |  |  |  |  |

**Supplementary Table 2.** **Immunological features of the patient**

|  | Age-matched normal value | Patient (1y) |
| --- | --- | --- |
| Blood cell counts |  |  |
| Leukocytes (10^9^/L) | [4-10] | 4.4 |
| Neutrophils (10^9^/L) | [1.7-8] | 0 |
| Eosinophils (10^9^/L) | [0-0.5] | 0.44 |
| Basophils (10^9^/L) | [0-0.2] | 0.02 |
| Monocytes (10^9^/L) | [0.2-1] | 0.52 |
| Lymphocytes (10^9^/L) | [1.5-4] | 3.2 |
| Hemoglobin (g/dL) | [12-17] | 11.7 |
| Platelets (10^9^/L) | [150-450] | 2.7 |
| T cells |  |  |
| CD3+ (%) | [69.0±9.0] | 84.7 % |
| CD4+ (%) | [60.7±7.3] | 59.5% |
| CD8+ (%) | [29.7±6.7] | 36.2 % |
| CD4/8 ratio | [0.9-2.6] | 1.6 |
| TCR α/β | [88.7±3.9] | 94.7 % |
| TCR γ/δ | [7.8±3.1] | 4.92 % |
| CD45RA+/CD4+ (naïve) | [75.9±8.5] | 69.6 % |
| CD45RA-CCR7+/CD4+ (central memory) | [41.9±11.7] | 33.5 % |
| CD45RA-CCR7-/CD4+ (effector memory) | [24.0±8.8] | 30.4 % |
| CD45RA+CCR7+/CD8+ (naïve) | [84.9±8.7] | 94.3 % |
| CD45RA-CCR7+/CD8+ (central memory) | [12.8±8.0] | 15.1% |
| CD45RA-CCR7-/CD8+ (effector memory) | [55.2±15.0] | 29.9 % |
| Treg (CD3^+^CD4^+^CD25^hi^CD127^low^) | [1.65±0.83] | 4.33 % |
| Th1 cells (% of CD3+CD4+CD45RO+) | [25.0±9.5] | 31.2% |
| Th2 cells (% of CD3+CD4+CD45RO+) | [41.4±10.6] | 39.7 % |
| Th17 cells (% of CD3+CD4+CD45RO+) | [22.0±6.2] | 21.9 % |
| TFH cells (% of CD3+CD4+) | [3.26±1.77] | 5.57 % |
| Activated T cells (% of CD3+CD4+) | [3.70±1.92] | 17.1 % |
| iNKT (% of CD3+) | [0.039±0.035] | 0.018 % |
| Recent thymic emigrants (% of CD3+CD4+ CD45RA+) | [88.0±8.6] | 90.7 % |
| TREC | [8.2 ± 6.3 ×10^2^ copies/ µg DNA] | NT |
| NK cells |  |  |
| CD56+CD16+ (% of lymphocytes) | [8.8±6.5] | 22.6 % |
| B cells |  |  |
| CD19+ (% of lymphocytes) | [16.1±7.4] | 8.80 % |
| CD19+CD27+ (memory) | [16.9±7.6] | 0.88 % |
| Immunoglobulins (Ig) |  |  |
| IgG (g/L) | [5.5-11.5] | 10.8 |
| IgA (g/L) | [0.4-1.6] | 0.65 |
| IgM (g/L) | [0.5-1.5] | 0.71 |
| Autoimmunity |  |  |
| ANA | <80 | <80 |
| Anti-dsDNA | < 10 | < 10 |
